# The Community Rehabilitation Assessment: patient and clinician-reported outcomes in ambulatory rehabilitation

**DOI:** 10.3389/fresc.2023.1123334

**Published:** 2023-05-22

**Authors:** Luke Andrew Turcotte, Caitlin McArthur, Charissa Levy, Rebecca Ho, John P. Hirdes, Katherine Berg

**Affiliations:** ^1^ Department of Health Sciences, Brock University, St. Catharines, ON, Canada; ^2^ School of Physiotherapy, Dalhousie University, Halifax, NS, Canada; ^3^ Rehabilitative Care Alliance, Toronto, ON, Canada; ^4^ Ontario Hospital Association, Toronto, ON, Canada; ^5^ School of Public Health Sciences, University of Waterloo, Waterloo, ON, Canada; ^6^ Department of Physical Therapy, University of Toronto, Toronto, ON, Canada

**Keywords:** ambulatory rehabilitation, patient-reported outcome measure, standardized health assessment, interRAI

## Abstract

**Objectives:**

The interRAI Community Rehabilitation Assessment (CRA) is a comprehensive health assessment designed to collect essential health and function information for rehabilitation care planning, benchmarking, and evaluation of clinic and home-based programs. A portion of the CRA is completed through patient self-report. The objective of this study was to demonstrate how the CRA can be used to describe the baseline clinical characteristics of patients participating in ambulatory rehabilitation programs and measure change across numerous domains of function, health, and wellbeing over time.

**Design:**

Cohort study.

**Setting and participants:**

In total, 709 patients were assessed with the CRA across 25 ambulatory clinics in Ontario, Canada between January 1st, 2018, to December 31st, 2018. We examined sub-groups of patients receiving rehabilitation following stroke (*n* = 82) and hip or knee total joint replacement (*n* = 210).

**Methods:**

Frequency responses and means were compared between admission and discharge from the ambulatory rehabilitation programs. Measures of interest included self-reported difficulty in completing instrumental activities of daily living, locomotion, fear of falling, and pain.

**Results:**

Significant improvement relative to at admission was detected for the overall cohort and both sub-samples on individual instrumental activities of daily living, stair difficulty, use of mobility aides, distance walked, fear of falling, and pain.

**Conclusions and implications:**

The standardized and comparable information collected by the CRA is expected to provide clinicians, clinic, and health system administrators with essential health and function information that can be used for care planning, benchmarking, and evaluation.

## 1. Introduction

Ambulatory, community, and home-based rehabilitation are effective and cost-efficient treatment modalities for individuals with functional impairment that may not require hospital-based care (1–3). In Ontario, Canada, ambulatory rehabilitation is delevered on an outpatient basis either directly in a hospital, or in another community clinic that is governed by a hospital (4). Ambulatory rehabilitation is recognized as an integral component of the continuum of care and is recommended for patients requiring post-discharge rehabilitation for conditions such as stroke (5) and total joint replacement (6). In 2013, the Auditor General of Ontario identified a need for the standardized data collection on the use ambulatory rehabilitation and associated patient outcomes (7).

In response, efforts to develop a minimum data set (i.e., a set of data elements for mandatory collection that are stored in a central repository) for ambulatory rehabilitation were undertaken, including a proof-of-concept pilot study by the Rehabilitative Care Alliance (RCA) to evaluate the feasibility of using a multidimensional health assessment to describe patient characteristics and outcomes (8, 9). The RCA is a provincially funded secretariat that works with partners across the province to strengthen and standardize rehabilitative care in Ontario through better planning, ongoing evaluation and quality improvement, and the integration of best practices across the care continuum.

The Community Rehabilitation Assessment (CRA) is designed to collect essential health and function information for use in clinic and home-based rehabilitation programs. It consists of two parts; a clinician and a patient self-report assessment, that can be administered at both the start and the end of an episode of care. By including both a patient-reported and clinician-completed component to the assessment, patients have an opportunity to communicate their needs with members of their rehabilitation team while simultaneously reducing clinician assessment burden. The CRA assessment serves as a patient-reported outcome measure of functional change and attainment of goals of care. It can also be used by health system administrators as a minimum data set to describe and compare patients at both system and clinic levels. Supplementary Material Table S1 provides an overview of the domains that are assessed by each portion of the assessment.

The CRA was developed by identifying items from of interRAI (https://interRAI.org) assessments for persons living independently in the community (10, 11), receiving home care (12–14), post-acute hospital (15) and residential long-term care (16) that are relevant to persons receiving care in ambulatory rehabilitative care. Items from the CRA can be used to calculate several validated interRAI summary scales, such as the ADL Long-form and Hierarchy Scale (17), Cognitive Performance Scale (18) and the Pain Scale (19). Patient ratings on these outcome scales can be compared directly with other interRAI assessments completed in other care settings (e.g., home care, long-term care, post-acute care, and rehabilitation) (20), which allows for seamless communication between sectors during transitions.

Most of the patient self-report items are shared with the interRAI Check-Up assessment and have demonstrated excellent to acceptable agreement with clinician-led assessments (10). The internal consistency and convergent validity of outcome scales based on self-report items are similar to interviewer and clinician-led assessments (11). As necessary, the patient self-reported assessment can be completed with the help of a family member or volunteer. When developing a care plan for rehabilitation, clinicians are encouraged to review and discuss responses on the self-reported assessment with the patient..

**Table 1:** Participant sociodemographic and clinical characteristics.

Characteristic	Overall Sample (*n* = 709)	Stroke (*n* = 82)	Total Joint Replacement (*n* = 210)
**Age (median, IQR)**	67 years (60–74)	68 years (57–76)	67 years (62–73)
**Gender**			
Female	55.8% (387)	39.7% (31)	64.3% (133)
Male	41.4% (287)	59.0% (46)	31.9% (66)
Other	2.9% (20)	1.3% (1)	3.9% (8)
**Marital Status**			
Married or partner/significant other	63.9% (441)	71.8% (56)	74.8% (154)
Unattached	36.1% (249)	28.2% (22)	25.2% (52)
**Lives Alone**			
No	75.4% (506)	74.0% (57)	80.3% (163)
Yes	24.6% (165)	26.0% (20)	19.7% (40)
**Employment Status**			
Employed	13.3% (91)	5.3% (3)	13.3% (27)
On leave	15.9% (109)	34.2% (26)	13.3% (27)
Seeking employment	3.6% (25)	1.3% (1)	3.0% (6)
Not seeking employment	67.3 (462)	59.2% (45)	70.4% (143)
**Primary Diagnosis**			
Total hip replacement	11.7% (83)	0	39.5% (83)
Total knee replacement	17.9% (127)	0	60.5% (127)
Stroke	11.6% (82)	100% (82)	0
Acquired brain injury	4.5% (32)	0	0
Spinal cord injury	3.7% (26)	0	0
Other orthopaedic condition	13.7% (97)	0	0
Other neurological condition	3.7 (26)	0	0
**Self-rated Overall Health**			
Excellent	11.7% (81)	13.0% (10)	15.7% (32)
Good	57.6% (398)	44.2% (34)	64.7% (132)
Fair	25.9% (179)	35.1% (27)	16.2% (33)
Poor	4.8% (33)	7.8% (6)	3.4% (7)
**Cognitive Performance Scale**			
0	73.4% (417)	37.5% (30)	90.5% (181)
1–2	24.5% (139)	55.0% (44)	9.0% (18)
3–4	1.6% (9)	6.25% (5)	0.0% (0)
5–6	0.5% (3)	1.25% (1)	0.5% (1)
**Activities of Daily Living Long Form Scale**			
0	70.8% (477)	58.8% (47)	61.4% (121)
1−2	14.0% (94)	15.0% (12)	21.8% (43)
3+	15.3% (103)	26.3% (21)	16.8% (33)
**Self-reported Mood Scale**			
0	45.2% (244)	41.5% (34)	47.2% (99)
1–3	29.4% (159)	24.4% (20)	27.1% (57)
4–9	25.4% (137)	34.2% (28)	25.7% (54)
**Fatigue**			
No	21.3% (147)	16.9% (13)	17.6% (36)
Yes, does not interfere with activities	46.7% (322)	45.5% (35)	43.9% (90)
Yes, interferes with activities	31.9% (220)	37.7% (29)	38.5% (79)
**Shortness of Breath**			
No	69.7% (480)	74.4% (58)	73.5% (150)
Yes, only during activities	27.7% (191)	23.1% (18)	24.5% (50)
Yes, at rest	2.6% (18)	2.6% (2)	2.0% (4)
**Self-reported Goal Attainment**			
All goals met	30.3% (164)	13.1% (8)	39.9% (69)
Progress in most areas	49.5% (268)	55.7% (34)	46.8% (81)
Progress in some areas	18.3% (99)	27.9% (17)	12.7% (22)
Little or no progress	1.9% (10)	3.3% (2)	0.6% (1)
Rehabilitation program length (median, IQR)	60 days (41–84)	63 days (44–88)	59 days (43–76)
Any physiotherapy sessions	94.8% (579)	88.8% (71)	100% (181)
Physiotherapy visits (median, IQR)^a^	8 visits (5–13)	10 visits (6–16)	9 visits (5–13
Any occupational sessions visits	31.6% (193)	83.8% (67)	5.6% (4)
Occupational sessions visits (median, IQR)^a^	9 visits (5–14)	9 visits (6–14)	5 visits (4–6)
Any speech-language pathology sessions visits	13.6% (83)	51.3% (41)	0% (0)
Speech-language pathology therapy sessions (median, IQR)^a^	7 visits (3–14)	9 visits (4–15)	0 visits (0–0)

^a^ Among patients with at least one visit for the provider.

Using data collected as part of a multi-clinic assessment process feasibility study of the CRA, the objective of this article is to illustrate the potential applications of the CRA as a minimum dataset for ambulatory rehabilitation clinics. We start by using the CRA to describe baseline patient characteristics across numerous domains of function, health, and wellbeing. Next, we demonstrate how information from the patient self-report and the clinician portions of the CRA can be used to compute interRAI summary scales and measure change in function and aspects of mobility over time.

## 2. Methods

### 2.1. Data source and participants

Twenty-five hospital and community-based ambulatory rehabilitation clinics in Ontario, Canada were recruited through an open call for participants by the RCA. Patients are referred to these publicly funded clinics by a physician or nurse practitioner. They must be either be aged (a) 65 years and older, (b) 19 years and younger, (c) recently discharged from hospital and require rehabilitation for the associated condition, illness, or injury, or (d) eligible for a social assistance program. Clinic treatment protocols, including duration and frequency, are at the discretion of the rehabilitation provider and based on individual patient need, goal attainment, and potential for improvement (21).

Like other interRAI comprehensive health assessments used as minimum data sets, the CRA is designed to be used with all ambulatory rehabilitation recipients. Thus, although participating clinics were asked to pre-commit to a minimum number of assessments for priority conditions such as hip or knee total joint replacement and stroke, the pilot study protocol did not specify patient exclusion criteria. Clinics were provided guidance on the suggested mode of administration of the self-report assessment for cognitively impaired patients. Interviewer assistance was indicated for patients with mild to moderate cognitive impairment (i.e., Cognitive Performance Scale (CPS) score of 2–3 (18)), and completion by and informal caregiver was indicated in the case of moderately severe or worse cognitive impairment (CPS 4+).

Participating clinics were encouraged to implement the CRA in a manner that was congruent with their current clinic workflow. Both patients and clinicians completed paper-based versions of the CRA on the first and last visit of treatment program. Regardless of impairment type, all patients and clinicians were instructed to complete all items on the assessment. Clinicians were instructed to assist patients to complete any unanswered self-report assessment items. In some clinics, volunteers and other staff members were available to assist patients that required assistance to complete the self-report portion of the assessment.

### 2.2. Statistical analysis

All analyses were performed for the overall sample and two sub-samples composed of individuals receiving rehabilitation following stroke (ICD-10-CA I64, I619, I634 and G459), and hip or knee total joint replacement (ICD-10-CA Z9660 and Z9661). We focused on these conditions because they represent distinct sub-populations in ambulatory rehabilitation that are part of Ontario's Health System Funding Reform model (5, 6).

Baseline patient characteristics, measured using information from both the patient and clinician responses on the assessment, were computed using frequency statistics for ordinal and nominal variables. Several interRAI summary scales can be computed using the information collected from the self-report and clinician rated portions of the assessment. We reported the Cognitive Performance Scale (range 0–6) (18) and the ADL Long Form Scale (range 0–28) (17) at baseline.

To demonstrate the CRA's utility as a measure of functional change, patient and clinician responses were compared between admission and discharge from the ambulatory rehabilitation program. Patients with missing data for a given assessment item on either assessment, most often because a discharge assessment was not completed, were omitted from the comparison. Scale scores were only calculated if all the required items for the scale were completed. Supplementary Material Table S2 compares the baseline clinical characteristics for patients where either a patient-self or clinician assessment was not completed.

Patient-reported difficulty on individual IADLs was measured using a 3-point response scale and compared between assessments using chi-square tests. This analysis was repeated for measures of mobility, including clinician-rated walking independence, self-reported stair difficulty, use of outdoor mobility aide, and farthest distance walked. Lastly, we compared clinician-rated timed 4-meter/13-foot walk test using paired *t*-tests.

Change in function was measured using the IADL Difficulty Scale at admission and discharge. This scale ranges from 0 to 14 points and is a sum of patient responses on the meal preparation, ordinary housework, managing finances, managing medications, phone use, shopping, and transportation items. Mean change between assessments was computed using paired *t*-tests. The Cohen's *d* statistic was also calculated to provide an effect size measure for the mean difference. Finally, we compared frequency distribution of the Pain Scale at admission and discharge.

## 3. Results

### 3.1. Patient demographics, health conditions, and goal attainment

A total of 709 patients were assessed with the CRA between January 1st, 2018, and December 31st, 2019. Four clinics submitted 50 or more admission assessments, nine clinics submitted 20–49 admission assessments and the remaining twelve clinics submitted fewer than 20 admission assessments.

The median age among participants in the sample was 67 years (IQR 60–74 years). Slightly more than half of the participants were female and almost two-thirds were married or had a partner/significant other. One-quarter of participants lived alone. Two-thirds of participants were retired or unemployed at admission, the majority of whom were not seeking work. A wide range of health conditions were represented in the sample, including patients receiving rehabilitation following total joint replacement (*n* = 210; knee *n* = 127, hip *n* = 83), stroke (*n* = 82), acquired brain injury (*n* = 32), spinal cord injury (*n* = 26), and other orthopaedic (*n* = 97) and neurological (*n* = 26) conditions (Table 1).

Approximately one-quarter of the total sample reported that their overall health at admission was “fair”, and an additional 4.8% reported that it was “poor”. Nearly three-quarters of the sample were cognitively intact at admission (CPS 0), with most of the other participants exhibiting mild cognitive impairment (CPS 1–2). Similarly, at admission, 70.8% of the sample were independent in all basic ADLs including personal hygiene, toilet use, locomotion and eating. Overall, 94.8% of participants received care from a physiotherapist, while only 31.6% and 13.6% of patients received care from an occupational and speech-language pathology therapists, respectively. The median number of sessions with each provider type ranged between 7 and 9 visits.

Overall, 557 patients (78.6%) completed a self-report discharge assessment, and for all but 15 patients (2.7%), an accompanying clinician discharge assessment was also completed. Clinicians completed discharge assessments in 30.9% of cases where a patient self-report discharge assessment was not completed. In cases where a discharge assessment was not completed, it was most often because the patient was discharged prematurely from rehabilitation due to a decline in health, the patient cancelled or failed to attend their final visit(s), or the rehabilitation program extended beyond the length of the study. Patients with a missing discharge self-report assessment were more likely to rate their overall health as “fair” or “poor”, report symptoms associated with depressive mood disorders, and experience shortness of breath at admission (Supplementary Material Table S2).

At discharge, nearly 80% of the overall sample reported that they achieved their rehabilitation goals or made progress in most areas. Compared to patients in the total joint replacement sub-group (39.9%), fewer patients in the stroke sub-group achieved all their rehabilitation goals (13.1%).

### 3.2. Change in functional Status following rehabilitation

**Figure 1:**
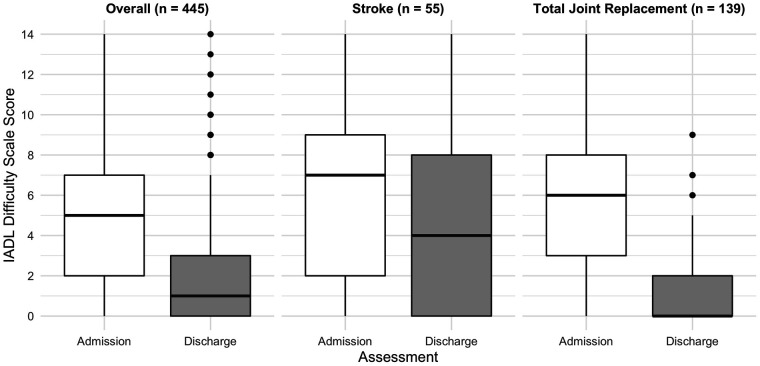
Distribution of instrumental activity of daily living difficulty scale score (0-14) at admission and discharge.

There was substantial improvement in IADLs. Relative to at admission, the percentage of patients in the overall sample and the stroke sub-group that reported some or great difficulty at discharge was significantly lower for all activities. For patients in the total joint replacement sub-group, this was only true for meal preparation, ordinary housework, medications, and shopping activities (Table 2). Among the overall sample, the mean improvement in the IADL Difficulty Scale (range 0–14) was 2.8 points (SD = 3.1, *P* < 0.001), which corresponds to a large effect size (Cohen's *d* = 0.9). This difference was small for the stroke sub-group (mean = 1.6 points, SD = 3.3, *P* < 0.001, Cohen's *d* = 0.4) and large for total joint replacement sub-group (mean = 4.3 points, SD = 3.2, *P* < 0.001, Cohen's *d* = 1.6). Figure 1 presents admission and discharge scores for this scale.

**Table 2:** Change in instrumental activity of daily living difficulty between admission and discharge.

		Overall Sample			Stroke			Total Joint Replacement		
IADL	Difficulty	Admission	Discharge	*P*-Value	Admission	Discharge	*P*-Value	Admission	Discharge	*P*-Value
Meal preparation	None	32.6% (169)	70.1% (364)	<0.01	33.9% (20)	52.5% (31)	<0.01	24.1% (39)	79.6% (129)	<0.01
	Some	45.3% (235)	24.3% (126)		37.3% (22)	28.8% (17)		46.9% (76)	19.1% (31)	
	Great	22.2% (115)	5.6% (29)		28.8% (17)	18.6% (11)		29.5% (48)	1.2% (3)	
Ordinary housework	None	23.7% (122)	56.9% (293)	<0.01	27.6% (16)	43.1% (25)	<0.01	14.5% (24)	66.7% (110)	<0.01
	Some	43.1% (222)	35.5% (183)		43.1% (25)	37.9% (22)		41.8% (69)	30.9% (51)	
	Great	33.2% (171)	7.6% (39)		29.3% (17)	19.0% (11)		44.0% (73)	3.0% (5)	
Managing finances	None	73.6% (365)	86.3% (428)	<0.01	48.2% (27)	60.7% (34)	<0.01	74.2% (115)	94.8% (147)	0.40
	Some	15.9% (79)	9.9% (49)		30.4% (17)	21.4% (12)		13.6% (21)	4.5% (7)	
	Great	10.5% (52)	3.8% (19)		21.4% (12)	17.9% (10)		12.3% (19)	0.7% (1)	
Managing medications	None	78.8% (408)	90.5% (469)	<0.01	53.5% (31)	70.7% (41)	0.02	81.7% (138)	97.6% (165)	<0.01
	Some	14.9% (77)	7.0% (36)		29.3% (17)	13.8% (8)		13.6% (23)	2.4% (4)	
	Great	6.4% (33)	2.5% (13)		17.2% (10)	15.5% (9)		4.7% (8)	0% (0)	
Phone use	None	89.3% (467)	94.5% (494)	<0.01	66.7% (40)	80.0% (48)	<0.01	94.0% (157)	98.2% (164)	1.0
	Some	7.5% (39)	4.8% (25)		26.7% (16)	15.0% (9)		2.4% (4)	1.8% (3)	
	Great	3.3% (17)	0.8% (4)		6.7% (4)	5.0% (3)		3.6% (6)	0% (0)	
Shopping	None	24.9% (128)	61.4% (316)	<0.01	31.1% (19)	49.2% (30)	<0.01	14.7% (24)	66.3% (108)	0.01
	Some	36.7% (189)	30.1% (155)		32.8% (20)	37.7% (23)		31.3% (52)	27.0% (45)	
	Great	38.4% (198)	8.5% (44)		36.1% (22)	13.1% (8)		54.0% (88)	6.7% (11)	
Transportation	None	38.3% (193)	72.6% (366)	<0.01	32.2% (19)	44.1% (26)	<0.01	25.3% (42)	81.8% (135)	0.2
	Some	22.6% (114)	16.1% (81)		22.0% (13)	20.3% (12)		24.1% (40)	13.3% (23)	
	Great	39.1% (197)	11.3% (57)		45.8% (27)	35.6% (21)		50.6% (84)	4.8% (8)	

### 3.3. Change in mobility following rehabilitation

Between admission and discharge, patients in the overall sample walked an average of 3.2 s faster (SD = 3.2, *P* < 0.0001) on the timed 4-meter/13-foot walk test, which corresponds with a medium effect size (Cohen's *d* = 0.6). Patients receiving rehabilitation following stroke improved by an average of 0.9 s (SD = 4.6, *P* < 0.001, Cohen's *d* = 0.2 (small effect size)) and patients receiving rehabilitation following total joint replacement improved by an average of 4.4 s (SD = 5.1, *P* < 0.001, Cohen's *d* = 0.8 (large effect size)).

At discharge, fewer patients in the overall sample and total joint replacement sub-group reported difficulty with stairs and need for a mobility aide when outdoors. Participants in the overall sample and both diagnosis sub-groups were able to walk further at discharge, with more than one-third of participants capable of walking more than one kilometer at discharge. Similarly, participants reported that they left their residence more frequently at discharge and that their fear of falling was reduced, such that they were able to engage in more activities. This was true for patients receiving rehabilitation for both stroke and total joint replacement. Fear of falling limited indoor walking and other activities at the same frequency at admission and discharge among the stroke sub-group (Table 3).

**Table 3:** Change in aspects of mobility between admission and discharge.

		Overall Sample			Stroke			Total Joint Replacement		
Measure	Response	Admission	Discharge	*P*-Value	Admission	Discharge	*P*-Value	Admission	Discharge	*P*-Value
Stair difficulty	None	35.1% (178)	53.1% (269)	<0.01	50.8% (31)	54.1% (33)	0.65	29.4% (47)	48.8% (78)	<0.01
	Some	41.8% (212)	38.7% (196)		26.2% (16)	29.5% (18)		46.9% (75)	46.9% (75)	
	Great	23.1% (117)	8.3% (42)		23.0% (14)	16.4% (10)		23.8% (38)	4.4% (7)	
Outdoor mobility aide	None	31.1% (172)	60.3% (323)	<0.01	43.6% (27)	54.8% (34)	0.54	7.3% (13)	59.9% (106)	<0.01
	Cane	23.3% (125)	19.8% (106)		12.9% (8)	12.9% (8)		39.6% (70)	30.5% (54)	
	Walker or crutches	36.6% (196)	14.7% (79)		27.4% (17)	17.7% (11)		48.6% (86)	9.0% (16)	
	Wheelchair	8.0% (43)	5.2% (28)		18.1% (10)	14.5% (9)		4.5% (8)	0.6% (1)	
Farthest distance walked	Did not walk	6.1% (32)	2.5% (13)	<0.01	8.3% (5)	1.7% (1)	<0.01	4.7% (8)	1.2% (2)	<0.01
	<5 meters	9.9% (52)	3.6% (19)		8.3% (5)	3.3% (2)		15.3% (26)	1.2% (2)	
	5–49 meters	25.8% (136)	10.1% (53)		18.3% (11)	10.0% (6)		31.8% (54)	13.5% (23)	
	50–99 meters	13.5% (71)	10.1% (53)		11.7% (7)	11.7% (7)		16.5% (28)	10.0% (17)	
	100–999 meters	28.1% (148)	36.2% (191)		30.0% (18)	38.3% (23)		25.9% (44)	35.3% (60)	
	1 + kilometers	16.7% (88)	37.6% (198)		23.3% (14)	35.0% (21)		5.9% (10)	38.8% (66)	
Fear of falling	Limits going outdoors	26.9% (136)	14.8% (75)	<0.01	24.6% (14)	14.0% (8)	<0.01	30.5% (50)	10.4% (17)	<0.01
	Of concern when walking in home	21.7% (106)	10.27% (50)	<0.01	16.1% (9)	16.1% (9)	0.01	21.9% (34)	6.5% (10)	<0.01
	Limits other activities	38.3% (187)	26.4% (129)	<0.01	28.6% (16)	28.6% (16)	<0.01	43.5% (67)	20.1% (31)	<0.01

### 3.4. Change in pain following rehabilitation

**Figure 2:**
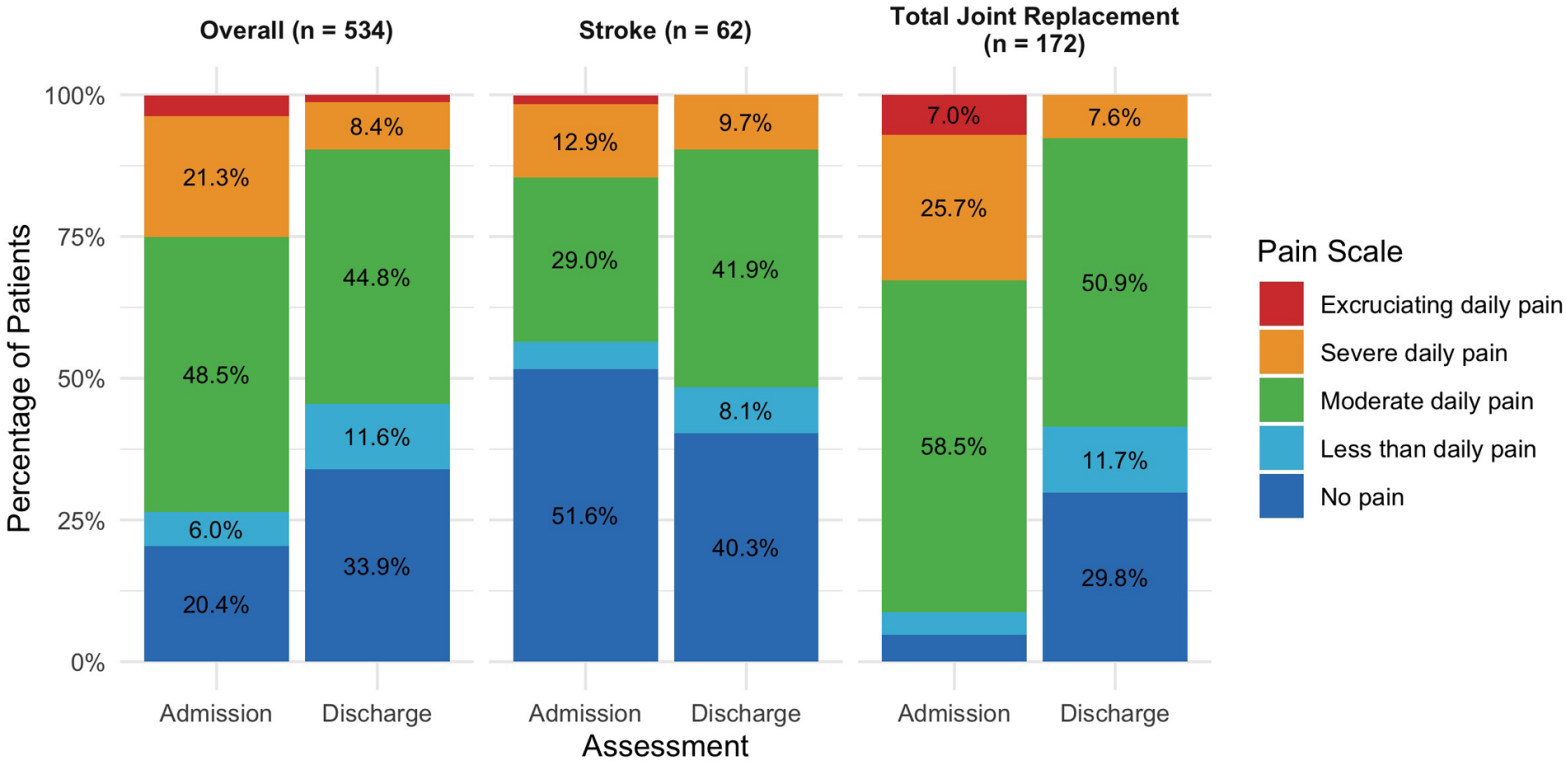
Pain scale distribution at admission and discharge. Value labels less than 5.0% suppressed.

At admission, 73.6% of the overall sample reported that they experienced daily pain compared to 54.5% of patients at discharge. Further, at admission, 25% of patients in the overall sample reported that the daily pain they experienced was severe or excruciating. The percentage of patients reporting pain at these intensities improved to 9.7% at discharge. Although patients in both the stroke and total joint replacement sub-samples experienced less frequent and intense pain at discharge, the difference was greatest for patients receiving rehabilitation following total joint replacement. For example, 32.7% reported severe or excruciating pain at admission compared to only 7.6% at discharge (Figure 2).

## 4. Discussion

Using information collected from both the patient and clinician perspectives, the CRA uses a novel approach to reduce clinician assessment burden while simultaneously capturing the patient's perspective of function, health, and wellbeing before and after rehabilitation. The sample of patients that participated in this assessment feasibility pilot study do not represent the Ontario ambulatory rehabilitation population at-large. However, as we have demonstrated in this article, the CRA has utility as a standardized health assessment that can be used to describe patient characteristics and functional outcomes in ambulatory rehabilitation clinics.

Increasingly, patient reported outcome measures are used to evaluate quality of care and complement clinical ratings (e.g., clinician-reported health status and adverse event rates) (22). The CRA is a patient-reported outcome measure that is augmented by clinician ratings in a subset of domains such as cognition, expression, and comprehension where patients that lack insight into their health may have difficulty responding accurately (23). At both organizational and system levels, standardized and comparable patient information will facilitate program planning and evaluation in similar ways as the mandated Resident Assessment Instrument (RAI) Minimum Data Set (MDS) 2.0, interRAI Long-term Care Facilities, and interRAI Home Care assessments used in Ontario and other Canadian provinces (24, 25).

The clinician and patient-rated portions of the CRA are designed to be used in tandem for care planning and evaluation. Clinicians are encouraged to review responses on the patient self-report portion of the assessment with the patient and to use it as a platform for discussions related to goals of care. Electronic assessment software was not available for this proof-of-concept pilot study but is typical for interRAI assessment implementations used in both facility and community-based settings. Tablet and computer-based assessments would allow clinicians to access outcome scales and other clinical decision-support algorithms for care planning purposes. Further, administration of the self-report component of the CRA through secure web portals would allow clinicians to gain an understanding of patient needs in advance of an initial visit.

The CRA is designed to assess individuals receiving rehabilitation for a broad range of health conditions, and therefore focuses on generic measures of function such as dependence in basic and instrumental ADLs and mobility. The advantage of generic activity and participation measures is that they allow comparison across conditions (26) and can be used to describe patients with multi-morbidity who do not fit conventional diagnosis groups. This allows for performance benchmarking between rehabilitation programs that differ with respect to patient population and severity of impairment (27). Methods of measuring clinic-level performance that account for differences in patient case-mix have not been developed yet for the CRA. However, rehabilitation-sensitive risk-adjusted quality indicators have been developed for interRAI assessments used in post-acute care (28), residential long-term care (29) and home care (30) settings. The CRA already measures most covariates used for indirect standardization for quality indicators related to change in ADLs, walking, gait, and pain such as cognitive performance, continence, and diagnosis. Since many patients in ambulatory rehabilitation programs are independent in ADLs, our quality indicator development efforts will also focus on measures of change in performance of IADLs.

Since the CRA is designed to collect a common core set of measures applicable across patient groups for use as a minimum data set, we expect that it will be complementary to other measures used by clinicians to assess condition-specific impairments (e.g., balance, range of motion, and strength) in ambulatory rehabilitation. For example, after identifying targets for interventions using condition-specific measures, the CRA may be used to evaluate the effects of those interventions, particularly on aspects of health-related quality of life (31). Future implementation studies should seek to understand clinician perspectives on the concurrent use of the CRA and other condition-specific measures. In addition to instrument refinement, this information can be used to develop education and training materials to maximize the clinical use the CRA and counteract perceptions that assessment is meant only to populate a minimum data set for program-level evaluation, oversight, and funding (32). To inform further refinement of the assessment, future studies should also measure aspects of assessment burden, including the time required to complete both components of the assessment, item difficulty, and need for assistance by a family member or other proxy. Finally, although the assessment items on the CRA have undergone psychometric testing in other dependent patient populations (10–19), future studies should evaluate the reliability and validity of these items among a broad range of patients (e.g., age groups, impairment types) receiving care for ambulatory rehabilitation.

## 5. Conclusions and implications

The CRA is a new multidimensional health assessment designed to be used in ambulatory and community-based rehabilitation clinics. Its two-part design allows it to be used as a general use patient-reported outcome measure and a clinician-rated measure of baseline function and change. The standardized and comparable information collected by the CRA is expected to provide clinicians, clinic managers, and health system administrators with valuable information for a range of applications including decision-support, care planning, planning, benchmarking, and evaluation.

## Data Availability

The participants of this study did not give written consent for their data to be shared publicly. Data access requests should be directed to Charissa.Levy@uhn.ca.
