# Supplementary material for: The Community Rehabilitation Assessment: patient and clinician-reported outcomes in ambulatory rehabilitation
Source: Front Rehabil Sci. 2023 May 22;4:1123334. doi: 10.3389/fresc.2023.1123334 (PMC10239873; doi:10.3389/fresc.2023.1123334)
Supplement: Supplementary file 1 [file Table1.docx]

Supplemental Table 1. Domains Assessed on the Self-report and Clinician-rated Portions of the Community Rehabilitation Assessment

| **Domain** | **Patient Self-report Assessment** | **Clinician-rated Assessment** |
| --- | --- | --- |
| Goals of care | Yes | No |
| Overall health | Yes | No |
| Sociodemographic (age, gender, marital status, employment) | Yes | No |
| Instrumental activities of daily living | Yes | No |
| Basic activities of daily living | Yes (bathing only) | Yes |
| Physical and social activity | Yes | No |
| Pain | Yes | No |
| Locomotion | Yes | Yes (gait speed only) |
| Falls | Yes | No |
| Health conditions (e.g., dyspnea, fatigue, dizziness) | Yes | No |
| Communication and vision | Yes | Yes |
| Mood | Yes | No |
| Memory and cognition | Yes | Yes |
| Diagnosis | No | Yes |
