# Supplementary material for: The Community Rehabilitation Assessment: patient and clinician-reported outcomes in ambulatory rehabilitation
Source: Front Rehabil Sci. 2023 May 22;4:1123334. doi: 10.3389/fresc.2023.1123334 (PMC10239873; doi:10.3389/fresc.2023.1123334)
Supplement: Supplementary file 2 [file Table2.docx]

Supplemental Table 2. Participant sociodemographic and clinical characteristics by completion of patient self-report and clinician discharge assessment

|  | **Patient Self-Report Discharge Assessment** | | **Clinician Discharged Assessment** | |
| --- | --- | --- | --- | --- |
| **Characteristic** | **Completed (n = 557)** | **Not Completed (n = 152)** | **Completed (n = 589)** | **Not Completed (n = 120)** |
| **Age (median, IQR)** | 68 (61 – 74) | 66 (54 – 73) | 67 (61-74) | 64 (54 – 74) |
| **Gender** |  |  |  |  |
| Female | 56.3% (309) | 53.8% (78) | 55.2% (319) | 58.6% (68) |
| Male | 41.4% (227) | 41.4% (60) | 42.6% (246) | 35.3% (41) |
| Other | 2.4% (13) | 4.8% (7) | 2.3% (13) | 6.0% (7) |
| **Primary Diagnosis** |  |  |  |  |
| Total Hip Replacement | 11.7% (65) | 11.8% (18) | 11.4% (67) | 13.3% (16) |
| Total Knee Replacement | 21.2% (118) | 5.9% (9) | 19.7% (116) | 9.2% (11) |
| Stroke | 11.3% (63) | 13.0% (19) | 12.9% (76) | 5.0% (6) |
| Acquired Brain Injury | 3.8% (21) | 7.2% (11) | 4.4% (26) | 4.2% (5) |
| Spinal Cord Injury | 3.6% (20) | 4.0% (6) | 3.6% (21) | 4.2% (5) |
| Other Orthopedic Condition | 14.4% (80) | 11.2% (17) | 13.9% (82) | 12.5% (15) |
| Other Neurological Condition | 3.2% (18) | 5.3% (8) | 3.6% (21) | 4.2% (5) |
| **Self-rated Overall Health** |  |  |  |  |
| Excellent | 11.4% (62) | 12.9% (19) | 11.3% (65) | 13.7% (16) |
| Good | 60.7% (330) | 46.3% (68) | 59.8% (343) | 47.0% (55) |
| Fair | 23.7% (129) | 34.0% (50) | 24.2% (139) | 34.2% (40) |
| Poor | 4.2% (23) | 6.8% (10) | 4.7% (27) | 5.1% (6) |
| **Instrumental Activity of Daily Living  Difficulty Scale (mean, SD)** | 4.9 (3.5) | 4.6 (3.5) | 5.0 (3.5) | 4.6 (3.4) |
| **Cognitive Performance Scale** |  |  |  |  |
| 0 | 74.7% (402) | 67.7% (92) | 73.3% (418) | 73.1% (76) |
| 1-2 | 23.2% (125) | 28.7% (39) | 24.6% (140) | 23.1% (24) |
| 3-4 | 1.5% (8) | 3.7% (5) | 1.6% (9) | 3.9% (4) |
| 5-6 | 0.6% (3) | 0% (0) | 0.5% (3) | 0% (0) |
| **Activities of Daily Living Hierarchy Scale** |  |  |  |  |
| 0 | 85.5% (458) | 83.8% (119) | 85.3% (587) | 84.1% (90) |
| 1-2 | 10.6% (57) | 9.2% (13) | 10.7% (61) | 8.4% (9) |
| 3-6 | 3.9% (21) | 7.1% (10) | 4.0% (23) | 7.5% (8) |
| **Self-reported Mood Scale** |  |  |  |  |
| 0 | 45.1% (246) | 40.3% (58) | 45.6% (262) | 36.5% (42) |
| 1-3 | 29.5% (161) | 22.2% (32) | 28.5% (164) | 25.2% (29) |
| 4-9 | 25.5% (139) | 37.5% (54) | 25.9% (149) | 38.3% (44) |
| **Fatigue** |  |  |  |  |
| No | 21.7% (118) | 20.1% (29) | 21.6% (124) | 20.0% (23) |
| Yes, does not interfere with activities | 48.1% (262) | 41.7% (60) | 47.6% (273) | 42.6% (49) |
| Yes, interferes with activities | 30.3% (165) | 38.2% (55) | 30.8% (177) | 37.4% (43) |
| **Shortness of Breath** |  |  |  |  |
| No | 72.2% (393) | 60.0% (87) | 71.1% (408) | 62.6% (72) |
| Yes, only during activities | 25.7% (140) | 35.2% (51) | 26.7% (153) | 33.0% (38) |
| Yes, at rest | 2.0% (11) | 4.8% (7) | 2.3% (13) | 4.4% (5) |
